# Assessment of Severity and Pattern of Early Childhood Caries Using ICDAS II Criteria: A Descriptive Cross-sectional Study

**DOI:** 10.31729/jnma.8782

**Published:** 2024-10-31

**Authors:** Amita Rai, Sunanda Sundas, Neha Dhakal, Anju Khapung

**Affiliations:** 1 Department of Pediatric and Preventive Dentistry, People's Dental College and Hospital, Nayabazar, Kathmandu, Nepal; 2 Department of Pediatric and Preventive Dentistry, Maharajgunj Medical Campus, Tribhuwan University Teaching Hospital, Maharajgunj, Kathmandu, Nepal; 3 Department of Community Dentistry, Maharajgunj Medical Campus, Tribhuwan University Teaching Hospital, Maharajgunj, Kathmandu, Nepal

**Keywords:** *early childhood caries*, *ICDAS II criteria;*, *pattern*, *severity*, *tooth type*

## Abstract

**Introduction:**

Early childhood caries is highly prevalent worldwide. The International Caries Detection and Assessment System incorporates both the non-cavitated and cavitated carious lesions with acceptable reliability. The objective of the present study was to assess the prevalence, severity and pattern of early childhood caries among children visiting outpatient department of a dental college in Nepal. The study also aimed to compare the severity and pattern based on individual tooth type.

**Methods:**

A cross-sectional study was conducted among 200 children of age group 24-71 months. The International Caries Detection and Assessment System II criteria was used to detect dental caries, and its severity and pattern. The ethical approval was taken from the institutional ethical committee (IRC Reference number: 1, 36, 2079/2080 IRC PDCH 2022 21).

**Results:**

Highest caries prevalence was seen among 48 to 60 month olds, whereas lowest prevalence was seen among the 24 to 35 month-old children. Based on severity, dentinal caries (Code 5) 526 (13.19%) was the most prevalent and undermined dentinal caries (Code 4) 36 (0.9%) was the least prevalent. The most common pattern of dental caries was multi-surface caries 644 (16.15%), and the least common pattern was the lingual surface caries 22 (0.55%). Prevalence of dental caries was highest in occlusal surface of mandibular second molars 151 (37.75%), and 227 (5.69%) of the teeth had received restoration and sealants of some sort (CARS).

**Conclusions:**

Majority of the children enrolled in the study had multiple cavitated and non-cavitated carious teeth involving different surfaces. Severity and pattern of caries also varied among individual tooth type.

## INTRODUCTION

Early childhood caries (ECC) is highly prevalent worldwide.^1,2,3^ Traditional system of dental caries detection, decayed, missing, and filled teeth (DMFT/dmft) index, fails to diagnose the non-cavitated carious lesions.^4,5^ The International Caries Detection and Assessment System (ICDAS) incorporates both the non-cavitated and cavitated carious lesions.^6^

The prevalence of ECC ranges from 9.3%-83% in the different parts of the world, and the prevalence among Nepalese children being towards the higher side.^7^ Cavitation of non-cavitated carious lesions can be prevented by timely diagnosis and prompt preventive management.^5^ Knowledge about severity and pattern facilitates in the planning and development of preventive and curative management for dental caries.^8^

There were limited studies regarding the assessment of severity and pattern of ECC using ICDAS criteria among Nepalese Children. Hence, the objectives of this study were to assess the prevalence, severity and pattern of ECC among 24 to 71-month-old children visiting People's Dental College and Hospital, Kathmandu, Nepal (PDCH), and to compare the severity and pattern of ECC of individual tooth type.

## METHODS

A cross-sectional study was conducted among 200 children of age group 24-71 months visiting the outpatient department (OPD) PDCH for dental treatment. The study was carried out over a period of six months (October 15, 2022 - April 15, 2023), and the convenience sampling method was used to select the study subjects. Before commencing the study, ethical approval was obtained from the Institutional Review Committee of People's Dental College and Hospital (Reference Number: 1, 36, 2079/2080 IRC PDCH 2022 21). Informed consent was obtained from the parents or the legal guardian before enrollment in the study. Children of age group 24-71 months with erupted complete sets of primary teeth, whose parents or legal guardians consented to participate in the study were included in the study. Whereas, children with special healthcare needs, and uncooperative children who could not open their oral cavity for dental examination without any assistance were excluded.

Sample size was calculated using the formula,

The formula used was as follows:


$$\begin{array}{ll} \text{n} & ={\text{Z}}^{2}\times \frac{\text{p}\times \text{q}}{{\text{e}}^{\text{2}}}\\ & =1.96^{2}\times \frac{\text{0.8525 }\times \text{ (100}-\text{0.8525)}}{0.05^{2}}\\ & =194\sim 200 \end{array}$$

Where:

n is the minimum required sample size for an infinite population.

Z represents the z-value (1.96) at a 95% Confidence Interval (CI).

p is the estimated prevalence of DH in the general population (85.25%).^9^

q = 1-p

e is the margin of error (5%)

Examination was performed by a single examiner. An intra-examiner reliability test was performed by examining a group of 25 children one week apart at two different time periods. Cohen's Kappa statistical analysis revealed that the Kappa coefficient for intraexaminer reproducibility was 0.84 for severity and 0.80 for pattern of caries.

A predesigned proforma was used for recording sociodemographic variables and dental caries status. The participants were seated on a dental chair and the teeth were cleaned using wet gauge. Under adequate illumination, all the surfaces of the teeth were assessed wet initially and air dried for five seconds which was then followed by re-examination. Each tooth was divided into five surfaces i.e., buccal, lingual, mesial, distal, occlusal/incisal to record the pattern of dental caries. Teeth with dental caries involving two or more than two surfaces and teeth with dental caries associated with restorations and sealants (CARS) were also recorded. The International Caries Detection and Assessment System (ICDAS) II criteria was used to detect the dental caries and its severity.^10-12^ In case of any confusion regarding proximal caries detection, bitewing radiographs were taken.

Data were entered in Microsoft Excel and then exported to and analysed using Statistical Package for Social Sciences (SPSS) version 20. For descriptive statistics, mean, standard deviation, and percentage were calculated.

## RESULTS

A total of 200 children were included in the study among which 99 (49.5%) were male and 101 (50.5%) were female. Early childhood caries was prevalent in all the study population of all age group (Table 1).

**Table 1:** Distribution of study (Early childhood Caries) population according to sex and age (n = 200).

Gender	Total n (%)	24-35 months	36-47 months	48-60 months	60-71 months
		n (%)	n (%)	n (%)	n (%)
Male	99 (49.50)	11 (36.67)	22 (55.00)	35 (52.24)	31 (49.21)
Female	101 (50.50)	19 (63.33)	18 (45.00)	32 (47.76)	32 (50.79)
Total	200 (100)	30 (100)	40 (100)	67 (100)	63 (100)

A total of 3988 teeth were examined as 12 teeth were missing due to dental caries or trauma in four children. The dental cries in children of age group 48-60 months was 1338 (33.46%) and dentinal caries (Code 5) was observed in 112 (8.37%) (Table 2).

Multi-surface caries was 644 (16.15%), and occlusal surface caries was 481 (12.06%). Among the study population, 227 (5.69%) of the teeth assessed were associated with restoration and sealants (CARS), (Table 3).

Enamel caries among the maxillary second molars (55/65) (Code 3) was in 94 (23.5%), and the dentinal caries (Code 5) in maxillary central incisors (51/61)was in 90 (22.78%) (Table 4).

Assessing the pattern/location of caries of individual tooth type, the occurance in maxillary central incisors 51/61 (multi-surface caries) was 162 (41.01%), occurance in occlusal surface of mandibular second molars (75/85) was 151 (37.75%) and that in maxillary second molars (55/65) was 144 (36%). (Table 5).

**Table 2:** Distribution of dental caries severity (n=3988).

Age	Total n (%)	Code 0 n (%)	Code 1 n (%)	Code 2 n (%)	Code 3 n (%)	Code 4 n (%)	Code 5 n (%)	Code 6 n (%)	CARS n (%)
24-35 months	597	337	27	39	62	2	62	35	33
	(100)	(56.45)	(4.52)	(6.53)	(10.39)	(0.34)	(10.38)	(5.86)	(5.53)
36-47 months	800	408	24	53	102	4	100	71	38
	(100)	(51)	(3)	(6.62)	(12.75)	(0.5)	(12.5)	(8.88)	(4.75)
48-60 months	1338	662	46	55	173	17	195	112	78
	(100)	(49.48)	(3.44)	(4.11)	(12.93)	(1.27)	(14.57)	(8.37)	(5.83)
60-71 months	1253	700	32	34	141	13	169	86	78
	(100)	(55.87)	(2.56)	(2.71)	(11.25)	(1.04)	(13.49)	(6.86)	(6.22)
Total	3988	2107	129	181	478	36	526	304	227
	(100)	(52.83)	(3.24)	(4.54)	(11.99)	(0.9)	(13.19)	(7.62)	(5.69)

**Table 3:** Distribution of dental caries pattern (n=3988).

Age	Total n# (%)	No caries n (%)	Buccal n (%)	Lingual n (%)	Mesial n (%)	Distal n (%)	Occlusal / Incisal n (%)	Two or more surfaces n (%)	CARS n (%)
24-35 months	597	337	58	3	11	2	69	84	33
	(100)	(56.45)	(9.71)	(0.5)	(1.84)	(0.34)	(11.56)	(14.07)	(5.53)
36-47 months	800	408	67	6	18	10	104	149	38
	(100)	(51)	(8.38)	(0.75)	(2.25)	(1.25)	(13)	(18.62)	(4.75)
48-60 months	1338	662	105	4	33	31	177	248	78
	(100)	(49.48)	(7.85)	(0.3)	(2.47)	(2.32)	(13.22)	(18.53)	(5.83)
60-71 months	1253	700	75	9	65	32	131	163	78
	(100)	(55.87)	(5.99)	(0.72)	(5.19)	(2.55)	(10.45)	(13.01)	(6.22)
Total	3988	2107	305	22	127	75	481	644	227
	(100)	(52.83)	(7.65)	(0.55)	(3.19)	(1.88)	(12.06)	(16.15)	(5.69)

**Table 4:** Dental caries severity of individual tooth type (n#= 200 and n=3988)

Tooth type	Total n (%)	Code 0 n (%)	Code 1 n (%)	Code 2 n (%)	Code 3 n (%)	Code 4 n (%)	Code 5 n (%)	Code 6 n (%)	CARS n (%)
55/65	400	140	11	20	94	9	64	24	38
	(100)	(35)	(2.75)	(5)	(23.5)	(2.25)	(16)	(6)	(9.5)
54/64	399	120	7	14	45	3	81	74	55
	(100)	(30.08)	(1.75)	(3.51)	(11.28)	(0.75)	(20.3)	(18.55)	(13.78)
53/63	399	252	29	42	32	1	42	1	-
	(100)	(63.16)	(7.27)	(10.52)	(8.02)	(0.25)	(10.53)	(0.25)	
52/62	397	172	26	32	50	2	77	34	4
	(100)	(43.32)	(6.55)	(8.06)	(12.6)	(0.5)	(19.4)	(8.56)	(1.01)
51/61	395	113	28	25	80	4	90	46	9
	(100)	(28.61)	(7.09)	(6.33)	(20.25)	(1.01)	(22.78)	(11.65)	(2.28)
75/85	400 (100)	104 (26)	10 (2.5)	11 (2.75)	67 (16.75)	6 (1.5)	60 (15)	87 (21.75)	55 (13.75)
74/84	400	142	8	11	50	8	81	37	63
	(100)	(35.5)	(2)	(2.75)	(12.5)	(2)	(20.25)	(9.25)	(15.75)
73/83	400	340	5	16	17	2	18	1	1
	(100)	(85)	(1.25)	(4)	(4.25)	(0.5)	(4.5)	(0.25)	(0.25)
72/82	398	362	1	5	20	-	8	-	2
	(100)	(90.96)	(0.25)	(1.26)	(5.02)		(2.01)		(0.5)
71/81	400	362	4	5	23	1	5	-	-
	(100)	(90.5)	(1)	(1.25)	(5.75)	(0.25)	(1.25)		
Total	3988	2107	129	181	478	36	526	304	227
	(100)	(52.83)	(3.24)	(4.54)	(11.99)	(0.9)	(13.19)	(7.62)	(5.69)

n#= total population, n = total tooth in 200 children

**Table 5:** Dental caries pattern of individual tooth type (n=3988).

Tooth type	Total n (%)	No caries n (%)	Buccal n (%)	Lingual n (%)	Mesial n (%)	Distal n (%)	Occlusal / Incisal n (%)	Two or more surfaces n (%)	CARS n (%)
55/65	400	140	11	2	8	-	144	57	38
	(100)	(35)	(2.75)	(0.5)	(2)		(36)	(14.25)	(9.5)
54/64	399	120	9	-	5	26	88	96	55
	(100)	(30.08)	(2.26)		(1.25)	(6.52)	(22.05)	(24.06)	(13.78)
53/63	399	252	89	7	8	13	3	27	-
	(100)	(63.16)	(22.3)	(1.76)	(2)	(3.26)	(0.75)	(6.77)	
52/62	397	172	73	7	34	2	1	104	4
	(100)	(43.33)	(18.39)	(1.76)	(8.56)	(0.5)	(0.25)	(26.2)	(1.01)
51/61	395	113	57	3	46	5	-	162	9
	(100)	(28.61)	(14.43)	(0.76)	(11.64)	(1.27)		(41.01)	(2.28)
75/85	400	104	10	1	4	-	151	75	55
	(100)	(26)	(2.5)	(0.25)	(1)		(37.75)	(18.75)	(13.75)
74/84	400	142	9	2	2	21	93	68	63
	(100)	(35.5)	(2.25)	(0.5)	(0.5)	(5.25)	(23.25)	(17)	(15.75)
73/83	400	340	32	-	6	6	1	14	1
	(100)	(85)	(8)		(1.5)	(1.5)	(0.25)	(3.5)	(0.25)
72/82	398	362	8	-	4	2	-	20	2
	(100)	(90.96)	(2.01)		(1)	(0.5)		(5.03)	(0.5)
71/81	400	362	7	-	10	-	-	21	-
	(100)	(90.5)	(1.75)		(2.5)			(5.25)	
Total	3988	2107	305	22	127	75	481	644	227
	(100)	(52.83)	(7.65)	(0.55)	(3.19)	(1.88)	(12.06)	(16.15)	(5.69)

## DISCUSSION

Early childhood caries is defined as "the presence of one or more decayed (non-cavitated or cavitated lesions), missing (due to caries), or filled tooth surfaces in any primary tooth" in a child under the age of six.^1^ Since the definition of ECC given by the American Academy of Pediatric Dentistry (AAPD) identifies both the non-cavitated and cavitated carious lesions as ECC, ICDAS can be considered as one of the best criteria to diagnose ECC. Studies have shown significant discrepancy in identification of carious lesions when the WHO criteria and ICDAS II criteria were compared.^5,6^

The prevalence of ECC has been shown to be variable in the different parts of the world.^7^ Studies done among Nepalese children in the community or among the children visiting dental hospitals for treatment have shown the prevalence of ECC to be very high. All the studies done among Nepalese children till date have used WHO criteria (deft index) to examine dental caries.^2,3,13^

In the present study, the most commonly affected teeth by ECC were mandibular second molars (75/85) (74%), and the least affected teeth were mandibular lateral incisors (72/82) (9.04%). Other studies done among the Nepalese children have shown similar pattern of dental caries distribution tooth wise.^3,13^ In another study done by Paucar et al. among the Colombian children using ICDAS II criteria, the highest and lowest prevalence of dental caries has been shown to be in the maxillary second molars and mandibular central incisors respectively.^8^ A study conducted by Henry et. al among 0-36-month-old Indian children showed that highest prevalence of dental caries was present in the maxillary central incisors and lowest prevalence was seen in the mandibular lateral incisors.^14^ The primary molar teeth are relatively late to erupt, whereas mandibular anterior teeth are early to erupt in the oral cavity. Contrary to the eruption timing, higher prevalence of dental caries in molars and lower in mandibular incisors indicates that lack of oral hygiene maintenance might be the cause of dental caries in the children, as maintaining cleanliness of posterior teeth is more difficult compared to the anterior teeth.^15^ This finding highlights the necessity to focus not only on the feeding practices but also in the oral hygiene maintenance to prevent ECC.

The results of the present study showed that non-cavitated lesions (Code 1, Code 2) were more prevalent among the maxillary anterior, and cavitated lesions (Code 3, Code 5, Code 6) were more common in the posterior teeth. Dentinal caries (Code 5) was the most frequent (13.19%) type based on severity which was followed by enamel caries (Code 3), non-cavitated carious lesions (Code 1 + Code 2), and extensive caries (Code 6). Contrary to the findings of the present research, study done in the community among Indian children by Bilal et. al. and among Brazilian children by Melgar et. al showed that non-cavitated carious lesions (Code 1 + Code 2) were more prevalent than the cavitated carious lesions.^16,17^ Since the present study was conducted among the children visiting dental hospital, the higher prevalence of cavitated lesions depicts that parents seek dental treatment for their children once the cavitation is noticed in their child's tooth/teeth. This highlights the need to focus on the preventive strategies which facilitates for the remineralization of the non-cavitated carious lesions, thus breaking the chain of dental caries progression.

Comparing the dental caries distribution according to the location of dental caries, multi-surface caries was the most prevalent pattern, which was then followed by occlusal surface and labial surface caries. The lowest prevalence based on pattern was lingual surface caries. The study conducted among the Colombian children has shown that the prevalence of dental caries was highest in the occlusal surfaces of molar teeth, and the lowest in the distal surfaces of mandibular central incisors and canines.^8^ Contrary to the findings of the present study, a study conducted by Henry et. al among Indian children in the community showed that buccal/lingual surface caries was the most common which was then followed by occlusal and mesial/distal surface caries.^14^ In the present study, prevalence of smooth surface caries was shown to be higher in the anterior teeth, whereas occlusal surface caries was shown to be higher in the posterior teeth. A study by Paucar et. al also showed that the prevalence of caries in the occlusal surfaces of molar teeth and labial surfaces of the anterior teeth was significantly higher as compared to the other surfaces.^7^ This pattern of dental caries distribution reflects inappropriate feeding practice and inadequate oral hygiene maintenance as the main cause of ECC.^18^ These findings highlight the need to focus on the preventive measures like education on feeding practices, oral hygiene instructions, diet counselling, fluoride application and pit and fissure sealants in the needful teeth, along with restorative treatment on the cavitated teeth.

Apart from studying the pattern and severity, there are various other factors related to ECC which needs to be studied in detail. Factors like methods of feeding, feeding of other food apart from breast milk, duration and frequency of breast feeding, whether or not water was given during or after feeding, caries experience in mothers, ethnicity, access to dental services etc. have shown very significant role in the occurrence of ECC.^2,3,17-20^ A study by Datta et. al conducted in 2020 has shown significance association between presence of active carious lesions and oral hygiene practice, feeding/dietary practice, dental attendance pattern and oral hygiene status.^21^ Since the objectives of the present study was to determine the pattern and severity of ECC based on ICDAS II criteria and to generate the baseline data, the above-mentioned factors could not be studied.

## CONCLUSIONS

The present study revealed that majority of the children enrolled in the study had multiple carious teeth which were multi-surface in pattern, and extending to the depth of enamel, dentin and even pulp. Variation in dental caries severity and pattern was also seen when compared among the individual tooth types, but the numbers of teeth which had received treatment for dental caries was very less.
